# Neural basis of romantic partners’ decisions about participation in leisure activity

**DOI:** 10.1038/s41598-019-51038-7

**Published:** 2019-10-08

**Authors:** Sunghyon Kyeong, Hyojung Eom, Min-Kyeong Kim, Young Hoon Jung, Sunyoung Park, Jae-Jin Kim

**Affiliations:** 1 Institute of Behavioral Science in Medicine, Yonsei University College of Medicine, Seoul, Republic of Korea; 2 Brain Korea 21 PLUS Project for Medical Science, Yonsei University, Seoul, Republic of Korea; 3 Department of Psychiatry, Yonsei University College of Medicine, Seoul, Republic of Korea

**Keywords:** Computational neuroscience, Social neuroscience

## Abstract

Leisure activity is one of key ingredients for individual happiness and life satisfaction. Enjoying leisure activity with one’s partner can increase marital satisfaction. This study aimed to identify the neural basis of making decisions on participation in a leisure activity with one’s romantic partner as well as the relationship between leisure activity and satisfaction with life. Thirty-seven soon-to-be married heterosexual couples were participated in functional MRI while deciding participation in specific leisure activities in the individual, partner, with-friend, and with-partner conditions. We constructed analysis of variance models and investigated couple characteristics such as personality similarity, leisure activity matching rate, and spatial similarity in the bilateral frontoparietal network. The results showed decreased activity in the bilateral hippocampus during the task in the with-partner condition. Individual leisure activity was correlated with quality of life in males, whereas participation in leisure activity might require more cognitive loading on the dorsolateral prefrontal cortex in females. The leisure activity matching rate was correlated with courtship period, personality similarity, and spatial similarity of the right frontoparietal network during the task. These findings suggest that although there are different activation pattern in making decisions on leisure activity between romantic couples, spatial similarity of the partner’s social brain networks may be a marker that predicts how well the couple enjoys leisure activity together. In addition, our couples’ data analysis provides a scientific basis for the saying that romantic couples become more similar the longer they are together.

## Introduction

Family, work, and leisure activity have been identified as key ingredients for individual happiness and life satisfaction^1^. Among these, leisure activity reduces stress^2^, provides psychological well-being^3^, delays age-related cognitive decline^4^, and improves quality of life^5^. Patterns of leisure activity differ according to various factors, including an individual’s personality, subjective well-being, and gender. For example, satisfaction with leisure activity is positively correlated with extraversion and negatively correlated with neuroticism^6^. Larger participation rates in leisure activity are related to higher levels of well-being^7^. Boys tend to spend time in outdoor leisure activities, whereas girls spend more time in indoor leisure activities^8^. Even in adulthood, this gender difference appears in many types of leisure activity including walking for leisure^9^ and team sports^10^.

People enjoy leisure activity individually or with others, including romantic partners. The decision about whether to share a leisure activity with someone is also influenced by many factors. From the brain’s perspective, self-referential processing and theory of mind may be important for this kind of decision. Brain regions related to self-referential processing are activated when people think about themselves and others^11^. In particular, the medial prefrontal cortex plays a key role in dealing with stimuli that are strongly associated with self^12^. When people imagine activity with others, brain regions related to theory of mind are activated to predict others’ behavior or preferences^13^. People have the ability to anticipate the behavior of others, almost as if they had read their minds. Together with the medial prefrontal regions, the temporoparietal junction has been consistently found to be involved in theory of mind ability in several previous studies^14^. For example, a previous study has shown that decisions for oneself and for other distinctly recruit brain regions including the temporoparietal junction^15^.

Meanwhile, when exploring the brain base of deciding whether to share activity with others, it is necessary to take into account the fact that the romantic partner triggers a special brain reaction that is different from a simple other person. It has been reported that the romantic partners provoke love-typical neural activity in the prefrontal, parietal, and limbic regions^16,17^. Furthermore, people in love show increased functional connectivity between the medial prefrontal cortex and temporoparietal junction compared to people not in love^18^. These regions may be love-related neural markers and would be fundamental components when individuals think about leisure activities that they prefer or dislike. In addition, a decision about participation in a specific leisure activity may involve a decision-making process based on the valuation of the intrinsic reward to happiness. Previous neuroimaging studies have consistently reported that decision making engages various frontoparietal regions including the dorsolateral prefrontal cortex^19^, orbitofrontal cortex^20^ and parietal regions^21^.

Leisure activity may also be deeply associated with marital satisfaction. For example, marital satisfaction increases when a married couple enjoys their leisure activity together rather than individually^22–24^. In fact, high levels of marital satisfaction are associated with the degree of similarity between couples^25^. The similarity of personality between couples has the effect of promoting their relationship^26^. Therefore, the regularity with which soon-to-be married couples participate in favorite leisure activities can be an important factor in determining the future of their marriage. Since the members of the couple are basically different in gender, have different characters, and have grown up in different environments, their favorite leisure activities may also be different. Because a family is a group of people living together, soon-to-be married couples need to learn to share their leisure activities; this can be a good starting point for achieving happiness.

This study aimed to identify the neural basis of making decisions on participation in a leisure activity with the partner in a soon-to-be married couple and the relationship between leisure activity and satisfaction with life. Our research question was “How similar or different are the neural responses in soon-to-be married couples when they think about leisure activity enjoying individually or together and with their partner or friend?” In order to answer this question, we developed an experimental functional magnetic resonance imaging (fMRI) task asking about participation in various leisure activities in four experimental conditions: individual, partner, with-friend, and with-partner. Given that thinking about participation in leisure activity may activate brain regions related to self-referential processing, theory of mind, and decision making, we hypothesized that the frontoparietal regions including the medial prefrontal cortex, dorsolateral prefrontal cortex, orbitofrontal cortex, temporoparietal junction, and other parietal regions would be involved in decisions about leisure activity with one’s partner or friend in soon-to-be married couples. In addition, we expected that there would be gender differences between the couples in these regions, taking into account gender differences in leisure activity preferences, but also predicted that similar patterns of leisure activity in couples would be related to similar neural engagement during decision making regarding leisure activity. Furthermore, we assumed that because of romantic love between them, the with-partner condition would produce stronger involvements of these frontoparietal regions than the with-friend condition and additional love-typical involvements in the limbic cortex.

## Results

### Demographic information

Demographic information is summarized in Table 1. The average courtship period of the 37 couples was 30.5 ± 18.6 months, and mean days remaining before wedding was 71.4 ± 33.3 days. The mean age of male participants was approximately 2 years older than that of their partner (*T*_74_ = 2.50, *P* = 0.015). The PLS (*T*_74_ = 1.15, *P* = 0.356) and WHOQOL-BREF (*T*_74_ = −0.36, *P* = 0.718) scores were not significantly different between the male and female groups. In the IPIP, no significant differences were observed in the extraversion, openness, agreeableness, and conscientiousness scores (*P* > 0.05), but the neuroticism scores were significantly higher in the female group than the male group (*T*_74_ = −5.46, *P* < 0.001).

**Table 1:** Summary of demographic and behavioral scales and its statistical comparison between the male and female groups.

Variable	Male (n = 37)	Female (n = 37)	Two sample t-test	
	(Mean ± SD)	(Mean ± SD)	*T(df* = *74)*	*P*-value
Age	31.70 ± 4.25	29.38 ± 3.72	2.50	0.015
WHOQOL-BREF	72.97 ± 12.22	74.05 ± 13.43	−0.36	0.718
Passionate Love Scale	208.27 ± 25.36	202.00 ± 21.55	1.15	0.256
**International Personality Item Pool**				
Neuroticism	22.97 ± 6.20	31.46 ± 7.13	−5.46	<0.001
Extraversion	33.03 ± 6.47	33.19 ± 7.73	−0.10	0.922
Openness	34.35 ± 4.60	34.32 ± 6.40	0.02	0.983
Agreeableness	34.62 ± 4.79	35.68 ± 5.05	−0.92	0.360
Conscientiousness	34.30 ± 5.52	35.51 ± 5.03	−0.99	0.325

SD, standard deviation; WHOQOL-BREF, abbreviated version of the World Health Organization Quality of Life.

### Behavioral data analysis

**Figure 1:**
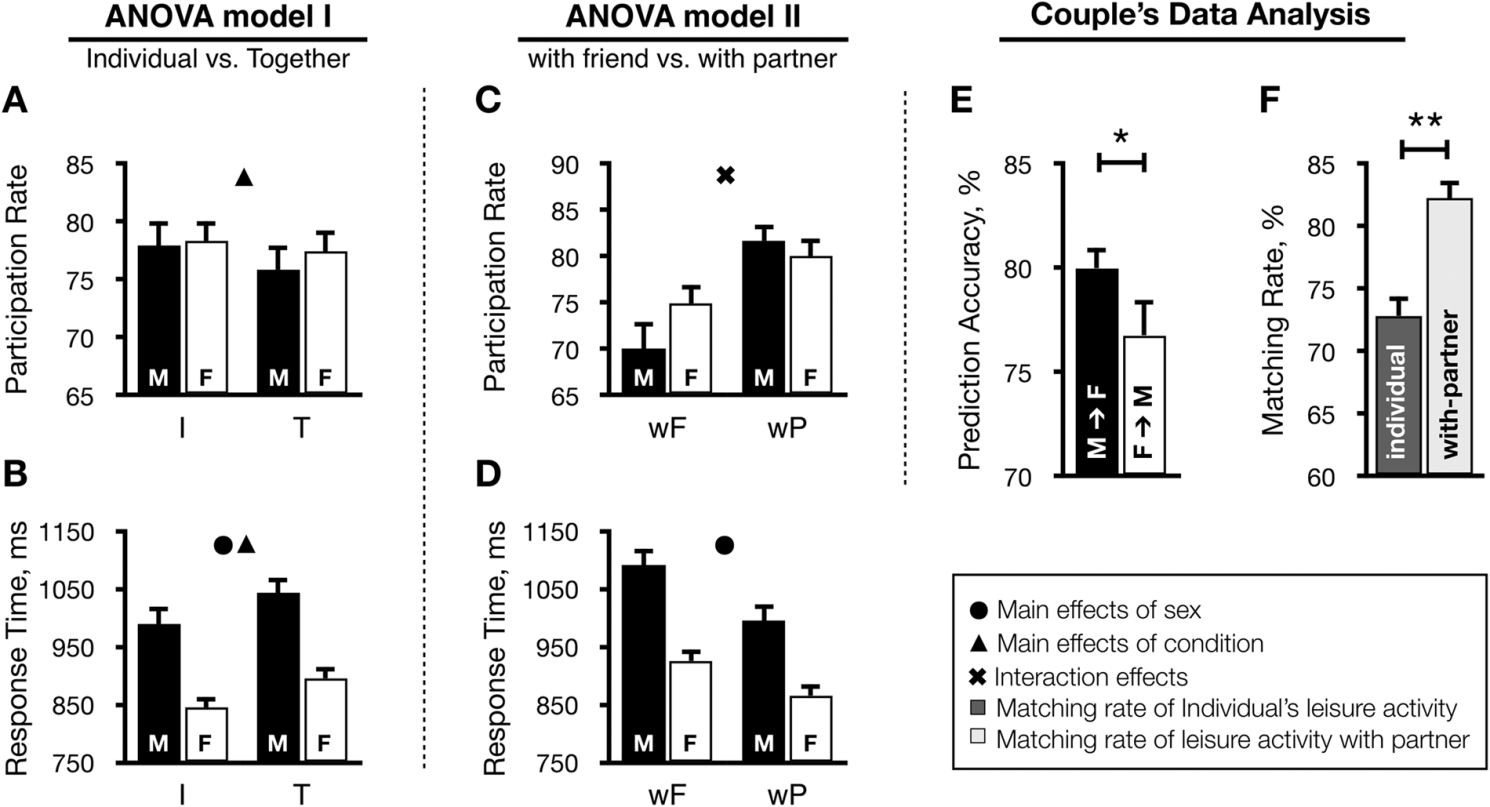
Behavioral responses showing participation rate and response time of leisure activity for analysis of variance (ANOVA) model I (**A**,**B**) and ANOVA model II (**C**,**D**), prediction accuracy of partner’s leisure activity (**E**), and leisure activity matching rate in the individual condition and with-partner condition (**F**). ANOVA model I: 2 (group, male and female) × 2 (condition, individual and together). ANOVA model II: 2 (group, male and female) × 2 (condition, with-friend and with-partner). Abbreviations: I, individual condition; T, together condition; wF, with-friend condition; wP, with-partner condition; M, male; F, female.

Figure 1 shows results from two ANOVA models and couple behavioral characteristics. In ANOVA model I, the leisure activity participation rate showed the significant main effect of condition (individual > together, *F*_1,71_ = 7.71, *P* = 0.007). The reaction time during the task showed significant main effects of sex (male > female, *F*_1,71_ = 17.17, *P* < 0.001) and condition (individual < together, *F*_1,71_ = 9.72, *P* = 0.003). In ANOVA model II, the leisure activity participation rate showed a marginally significant sex × condition interaction (*F*_1,71_ = 3.95, *P* = 0.051). The post-hoc analysis revealed that the male group preferred to perform leisure activity with their partner rather than their friends compared to the female group (*T*_72_ = 2.43, *P* = 0.018). The reaction time during the task showed a significant main effect of sex (male > female, *F*_1,71_ = 18.48, *P* < 0.001). In partial correlation analysis, the individual leisure activity participation rate showed a positive association with the WHOQOL-BREF score in the male group (*r* = 0.43, *P* < 0.01), but not in the female group. The with-partner and with-friend leisure activity participation rates showed no significant correlations with the PLS score.

### fMRI data analysis

**Figure 2:**
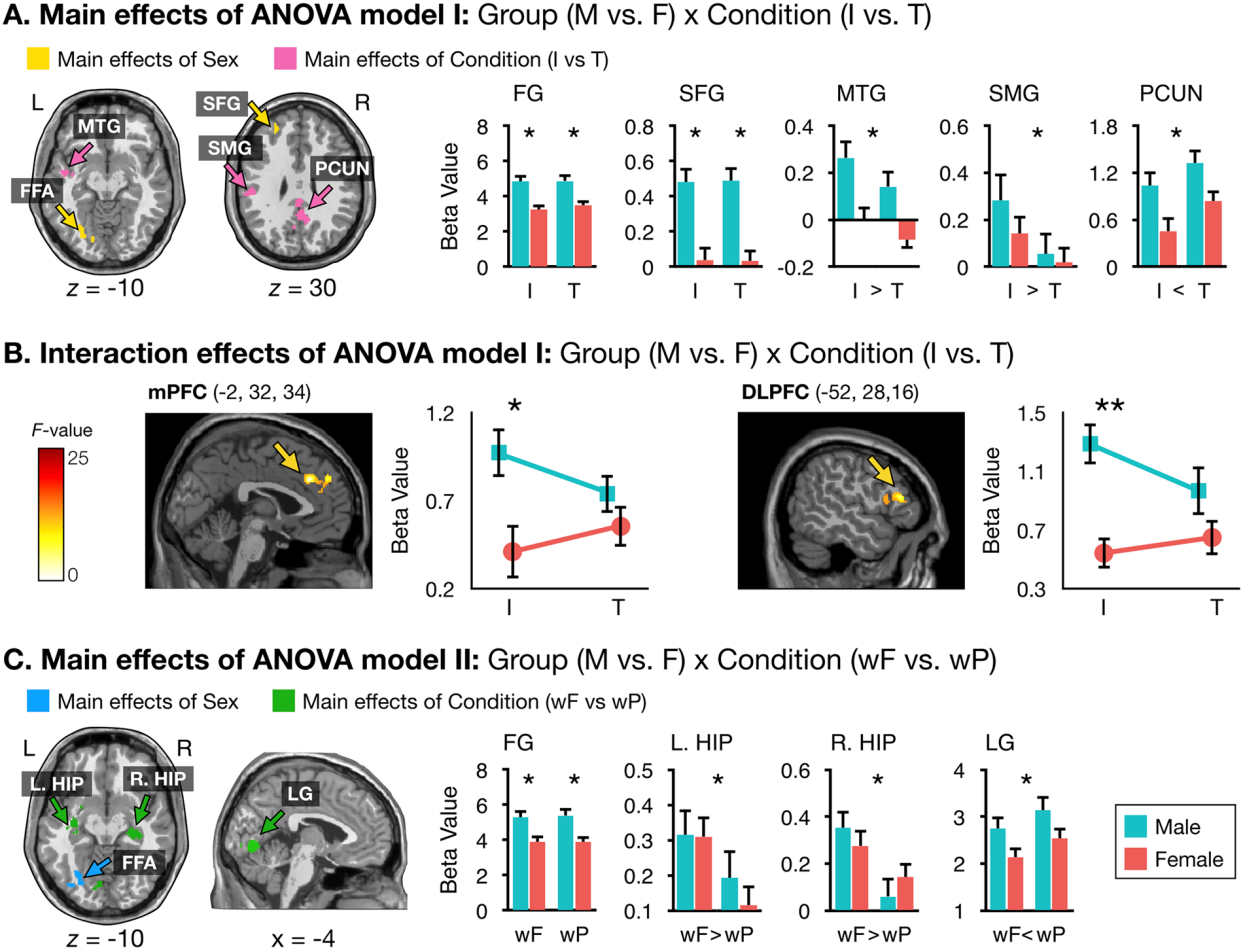
Neural responses showing main and interaction effects of ANOVA model I (**A**,**B**) and main effect of ANOVA model II. (**C**) Abbreviations: DLPFC, dorsolateral prefrontal cortex; FG, fusiform gyrus; HIP, hippocampus; I, individual condition; LG, lingual gyrus; mPFC, medial prefrontal cortex; PCUN, precuneus; SMG, supramarginal gyrus; SFG, superior frontal gyrus; MTG, middle temporal gyrus; T, together condition; wF, with-friend condition; wP, with-partner condition. *P < 0.05 for post-hoc comparisons.

The comparison between the individual and partner conditions and between the partner condition and the partner’s individual condition did not show any significant difference. However, two different ANOVA models revealed significant clusters, which are summarized in Table 2. Figure 2A presents examinations of group (male versus female) and condition (individual versus together) factors while conducting the leisure activity fMRI tasks. In ANOVA model I, the main effect of group was seen in the left superior frontal gyrus and left fusiform gyrus. The main effect of condition was observed in the left superior temporal gyrus, right precuneus, and left postcentral gyrus. The group × condition interaction effect was seen in the left medial prefrontal cortex and left dorsolateral prefrontal cortex. The results from post-hoc analysis on these regions characterizing the interaction are shown in Fig. 2B. Beta values in the left medial prefrontal cortex and left dorsolateral prefrontal cortex during decisions about participation in leisure activity in the together condition did not significantly differ between the male and female groups, whereas those in the individual condition were significantly higher in the male group compared to the female group (*P* < 0.05). The beta values in the brain regions identified in ANOVA model I showed no significant correlation with the WHOQOL-BREF scores. In correlation analysis between the regional beta values and leisure activity participation rates, only significant result was the negative correlation in the left dorsolateral prefrontal cortex in the individual condition in the male group (*r* = −0.56, *P* < 0.001).

**Table 2:** Significant clusters obtained from two different ANOVA models.

Region	MNI coordinate, mm			Nvox	Zmax	P_FWE_	Post-hoc comparison
	x	y	z				
**ANOVA model 1: Group (male vs**. **female)** × **Condition (individual vs**. **together)**							
Main effect of group							
L. Fusiform gyrus	−14	−78	−14	179	4.78	0.007	male > female
L. Superior frontal gyrus	−22	44	20	120	4.12	0.043	male > female
Main effect of condition							
L. Middle temporal gyrus	−48	−6	−12	285	5.11	<0.001	individual > together
R. Precuneus	0	−48	36	533	4.7	<0.001	individual < together
L. Supramarginal gyrus	−52	−24	32	264	4.86	0.001	individual > together
Interaction							
L. Medial prefrontal cortex	−2	32	34	183	4.62	0.007	see Fig. 2B
L. Dorsolateral prefrontal cortex	−52	28	16	145	4.22	0.02	see Fig. 2B
**ANOVA model 2: Group (male vs**. **female**) × **Condition (with-friend vs**. **with-partner)**							
Main effect of group							
L. Fusiform gyrus	−34	−78	−14	138	3.87	0.023	male > female
Main effect of condition							
L. Hippocampus	−32	−12	−8	273	4.67	0.001	with-friend > with-partner
R. Hippocampus	36	−22	−12	193	5.93	0.005	with-friend > with-partner
L. Lingual gyrus	−4	−76	−6	287	5.15	<0.001	with-friend < with-partner
Interaction							
Not significant							

P_FWE_, cluster-level family-wise error-corrected p-value; L., left; R. right.

Figure 2C shows examinations of group (male versus female) and condition (with-friend versus with-partner) factors while performing the leisure activity fMRI tasks. In ANOVA model II, the main effect of group was seen in the left fusiform gyrus. The main effect of condition was observed in the bilateral hippocampus and left lingual gyrus. The group × condition interaction effect was not observed. In correlation analysis between the regional beta values and PLS scores, only significant result was the negative correlation in the left hippocampus in the with-partner condition in the male group (*r* = −0.42, *P* = 0.001). The regional beta values showed no significant correlation with the leisure activity participation rates.

### Group independent component analysis and bilateral frontoparietal networks

We selected two independent components matched to the left and right frontoparietal networks, respectively. The left frontoparietal network included the left prefrontal cortex, dorsomedial prefrontal cortex, and left superior parietal lobule. The right frontoparietal network included the right prefrontal cortex, dorsomedial prefrontal cortex, and right superior parietal lobule. Table 3 shows the results from the statistical comparison of functional connectivity (FC) of the bilateral frontoparietal networks between the male and female groups. Compared with the female group, the male group exhibited significantly increased functional connectivity between the left frontoparietal network and left ventrolateral prefrontal cortex and between the right frontoparietal network and left cerebellum and significantly decreased functional connectivity of the left frontoparietal network with the left dorsolateral prefrontal cortex, left orbitofrontal cortex, left anterior cingulate cortex, and right precentral gyrus.

**Table 3:** Statistical comparisons of functional connectivity between the male and female groups.

Functional connectivity		MNI coordinate, mm			Nvox	Zmax
Source	Target	x	y	z		
**Male** > **Female**						
L. FPN	L. Frontopolar cortex	−30	58	10	120	4.78
R. FPN	L. Cerebellum	−12	−76	−22	128	4.87
**Male** < **Female**						
L. FPN	L. Dorsolateral prefrontal cortex	−26	34	40	155	3.94
L. FPN	L. Orbitofrontal cortex	−30	60	−12	108	4.33
L. FPN	L. Anterior cingulate cortex	−4	46	10	177	4.81
L. FPN	R. Postcentral gyrus	58	−10	20	103	4.85

The left frontoparietal network (L. FPN) and right frontoparietal network (R. FPN) were extracted using the dual-regression approach.

### Couples’ data analysis

A paired sample t-test revealed that partners’ leisure activity matching rates in the with-partner condition were significantly higher than those in the individual condition (*T*_36_ = 6.2, *P < *0.005). In evaluating the prediction for the partner’s leisure activity by matching the responses between a participant’ partner condition and his/her partner’s individual condition, the male group had a higher prediction accuracy of partner’s leisure activity than the female group (*T*_36_ = 2.0, *P* = 0.051). The prediction accuracy showed a significant negative correlation with the WHOQOL-BREF scores in the female group (*r* = −0.37, *P* = 0.023), but not in the male group. In contrast, it showed no significant correlation with the PLS scores in either the female or male group. Courtship period showed a significant positive correlation with the leisure activity matching rate in both the individual (*ρ* = 0.36, *P* = 0.031) and together (*ρ* = 0.49, *P* = 0.003) conditions.

**Figure 3:**
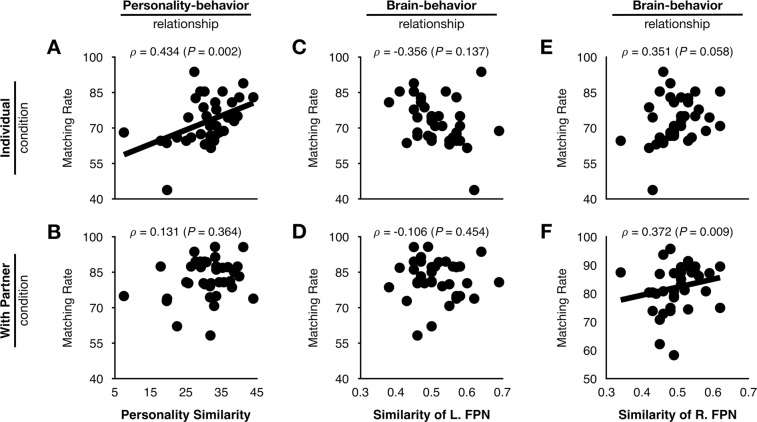
Associations of leisure activity matching rate with personality similarity (**A**,**B**) and spatial similarity of the left frontoparietal network (**C**,**D**) and right frontoparietal network (**E**,**F**) in the individual and with-partner conditions.

**Figure 4:**
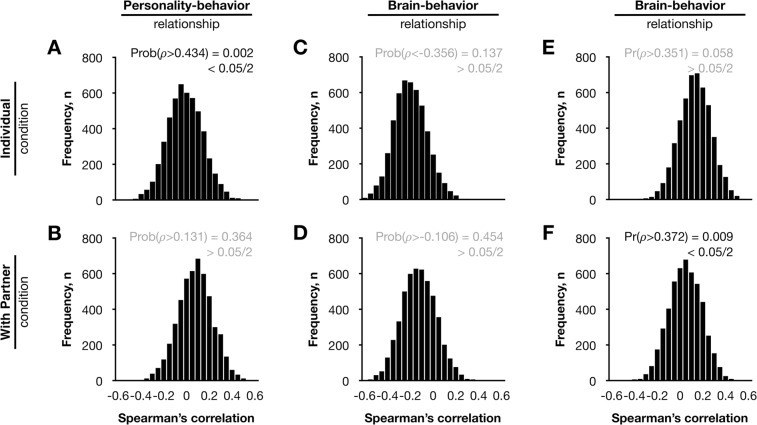
Distribution of couple characteristics obtained from random couples. Diagrams show distribution for personality similarity and matching rate of leisure activity (**A**,**B**), distribution for matching rate of leisure activity and similarity of spatial pattern of the left frontoparietal network (**C**,**D**), and distribution for the similarity of spatial pattern of the right frontoparietal network (**E**,**F**).

Figure 3 shows couple characteristics such as the personality-behavior relationship and the brain-behavior relationship, and their significance was computed from the null distribution in the random male-female samples (Fig. 4). In the personality-behavior relationship, personality similarity and the leisure activity matching rate showed a significant positive correlation in the individual condition, which was not observed in the random pairs (*ρ*_couple_ = 0.434, Prob(*ρ* > 0.434) = 0.002). These two variables showed no significant correlation in the with-partner condition, and this feature was similar in the random pairs (*ρ*_couple_ = 0.131, Prob(*ρ* > 0.131) = 0.364). In the analysis of partners’ brain-behavior relationships, similarity of the right frontoparietal network and the leisure activity matching rate showed a significant positive correlation in the with-partner condition, which was not observed in the random pairs (*ρ*_couple_ = 0.372, Prob(*ρ* > 0.372) = 0.009). The brain-behavior relationships of the leisure activity matching rate with similarity of the left frontoparietal network in the individual and with-partner conditions and with similarity of the right frontoparietal network in the individual condition were not significant in that the significance values were similar to those in the random pairs (*ρ*_couple_ = −0.356, Prob(*ρ* < −0.356) = 0.137; *ρ*_couple_ = −0.106, Prob(*ρ* > −0.106) = 0.454; *ρ*_couple_ = 0.351, Prob(*ρ* > 0.351) = 0.058, respectively).

## Discussion

In this study, we measured the personality-behavior relationship and brain-behavior relationships of soon-to-be married couples to understand their characteristics during decisions about participation in leisure activity. For this purpose, we used an fMRI task regarding decisions about participation in specific leisure activities in the individual, partner, with-friend, and with-partner conditions and analyzed couple’s brain characteristics related to leisure activity. Our soon-to-be married couples were wildly or passionately in love as estimated with the PLS and were found to be highly satisfied with their lives as assessed with the WHOQOL-BREF. Although they share many parts of their lives because they love each other and this sharing can be important to keep them highly satisfied with their lives, it is not easy to share leisure activity fully because of the differences in living together. Understanding the similarity and difference in brain characteristics during decisions about participation in leisure activity can provide useful clues in expanding the sharing of leisure activity.

The results form ANOVA model I showed different neural activity in the temporoparietal regions between the together and individual conditions during decisions about participation in leisure activity. The hypothesis predicted the difference in the frontoparietal regions, but the actual results were found in the temporoparietal regions. In particular, the precuneus was significantly increased in the together condition relative to the individual condition. From the neuroscientific point of view, decisions about participation in a specific leisure activity require retrieval of episodic memory if the individual has experienced that leisure activity in the past. Episodic memory allows individuals to engage in mental travel into both the past and the future^27^. Given that the precuneus plays a role in visual imagery of episodic memory content^28^ and egocentric projection of episodic autobiographical memory^29,30^, our finding in the precuneus suggests that individual’s explicit memory, such as autobiographical and episodic memory was used more in the together condition than in the individual condition. This is probably because whether to do a leisure activity alone is an immediate judgment of an individual’s preferences, whereas whether to do it with someone requires explicit memory of the individual at the base of the judgment.

On the other hand, neural activity in the left middle temporal gyrus and left supramarginal gyrus was significantly increased in the individual condition relative to the together condition, suggesting that these two regions may play some roles in the immediate judgment of an individual’s preferences. This judgment may be based on a simple high concentration or imitating imagination of our experimental stimulus under the individual condition, considering that the middle temporal gyrus is involved in processing the audiovisual speech^31^ and the supramarginal gyrus, part of the mirror neuron, is engaged in gestural production^32^ and manipulation knowledge^33^. Meanwhile, individual leisure activity participation rates were positively correlated with the WHOQOL-BREF scores in the male group, but not in the female group. Given that engagement in leisure activity contributes to subjective well-being^34^, this result implies that higher leisure activity participation rates in the individual condition are associated with higher subjective life satisfaction in the male participants. Although men and women showed different leisure activity patterns, soon-to-be married couples will share much of their future lives and understanding one’s partner’s leisure activity patterns is important for marital satisfaction. We observed that a longer courtship period was positively associated with similarity of leisure activity pattern in soon-to-be married couples. These results support a common belief that couples become increasingly similar in their appearance and behavior^35,36^.

Soon-to-be married couples showed higher leisure activity participation rates in the with-partner condition than in the with-friend condition. Considering that enjoying leisure activity with one’s partner is related to high marital satisfaction^22–24^ and essential to maintain relationships^37^, our results suggest that these couples have a good chance of happy marital life. In our results from ANOVA model II, neural activity in the bilateral hippocampus during decisions about participation in leisure activity was significantly decreased in the with-partner condition relative to the with-friend condition. The medial temporal regions including the hippocampus are known to be pivotal in integrating information from past experience^38^. Our result in the hippocampus may imply that even within the same together condition, episodic memory may be much less used in the with-partner condition than in the with-friend condition. This is in line with our hypothesis that the with-partner condition would produce a love-typical involvement in the limbic regions. We presume that this feature in romantic couples is due to unconditional love between the two rather than their own episodic memory in making decisions regarding leisure activity with their partners. This may be supported by another finding that neural activity in these regions showed a negative correlation with the PLS scores in the male group. If an individual does not have sufficient affection for his/her partner, deciding to do something together can be a coping strategy to just avoid the conflict. Previous studies have shown that the hippocampus is sensitive to approach-avoidance conflicts and anxiety related to decision-making situations^39^. In the present study, hippocampal activity in male participants who expressed fewer feelings of love tended to be higher. This might be related to their anxiety about the conflict processing when deciding to do something with their partner^40^. Meanwhile, neural activity in the lingual gyrus during decisions about participation in leisure activity was significantly increased in the with-partner condition relative to the with-friend condition. Considering that the lingual gyrus is involved in processing mental imagery^41^ and activation of this region has been observed when high-emotional stimuli are given^42^, it can be assumed that intense emotions may affect the imagination of leisure activity with a romantic partner.

Our imaging analysis showed the sex × condition (individual vs. together) interaction effect. In the post-hoc test, when compared to men, women showed decreased neural activity in the medial prefrontal cortex and left dorsolateral prefrontal cortex during decisions about individual participation in leisure activity, suggesting that the task may require less cognitive effort in women than in men. Women tend to be more intuitive than men, and intuitive thought is processed automatically and unconsciously and requires little cognitive effort^43^. The faster reaction time in women in our behavioral results may also be related to their intuitive decision making. It is worth noting that life satisfaction may affect neural activity in the medial and lateral prefrontal regions for positive or negative emotion^44^. Considering that the cortical midline structure including the medial prefrontal cortex is involved in evaluation of self-referential statements^12^, our fMRI task regarding decisions about participation in individuals’ leisure activity may be associated with provocation of self-referential processing. In addition, the strength of neural activation in the dorsolateral prefrontal cortex was negatively correlated with individual’s quality of life scores in the female group, suggesting that decisions about participation in leisure activity might require more cognitive loading in females with lower scores for quality of life.

Gender differences in behavioral and neural responses have been observed in many domains, including episodic memory^45^, emotional memory^46^, language processing^47^, and emotional responses^48^. In our study, men showed slower responses during decisions about participation in leisure activity than women. Since our ANOVA models included age as a covariate, faster response in the female group would not be explained by age differences between the groups. Interestingly, men showed higher prediction accuracy of their partner’s leisure activity than women. Given that men make decisions slower^49,50^ and score better on performance prediction^51^ in behavioral tasks than women, our results suggest that women might have a superior perceptual performance in decisions about participation in leisure activity and men might show a superior cognitive performance in predictions of their partners’ leisure activity.

To test the third hypothesis that similar patterns of leisure activity in soon-to-be married couples would be related to similar neural engagement during decision making regarding leisure activity, we first observed personality-behavior relationships in the couples. Specifically, the more similar are the personalities of the partners in a couple, the more similar their favorite leisure activities are likely to be. Importantly, these personality-behavior relationships were not observed in randomly paired couples. In a previous study, leisure activity was positively correlated with extraversion and conscientiousness and negatively correlated with neuroticism^52^. In fact, individual differences in personality have been associated with functional connectome, and these differences have been related to preference for inhibited, passive, and inactive behavior^53^. Considering that enjoying leisure can be a major source of satisfaction with life and that personality is related to overall life satisfaction^54^, our results indicate that the greater correlation between the personality similarity and leisure activity matching rate might be associated with future marital satisfaction of soon-to-be married couples.

Gender differences in neural responses during the task were also observed in functional connectivity. We observed multiple regions showing significant differences in functional connectivity of the left frontoparietal network between the male and female groups, but not in that of the right frontoparietal network. In brain-behavior analysis, the leisure activity matching rate in the with-partner condition showed a positive relationship with the similarity of the right frontoparietal network between the couples. However, a similar relationship was not observed in the left frontoparietal network. It is worth noting that the functional role of the frontoparietal network, which includes the lateral and medial prefrontal cortex and the temporal-parietal areas, has been lateralized in several studies. For example, the left dorsolateral prefrontal cortex has been linked to a cognitive control and task performance^55^, whereas the right dorsolateral prefrontal cortex and right inferior parietal regions have been associated with theory of mind in the aspects of pain perception^56^, visual perspective taking^57^, and reasoning^58^, respectively. Considering that our task required participants to predict a partner’s choice, the neural system related to theory of mind might be involved during decisions about participation in leisure activity in the with-partner condition. Taken together, our results on the spatial similarity of couples’ social brain networks (i.e., the right frontoparietal network) suggest that couples become more similar in their brain-behavior relationships.

There are some limitations in our study. First, because men get married about three years later than women in Korea, on average^59^, we could not recruit age-matched couples. Therefore, the age differences between men and women were controlled in the statistical analysis, and we tried to account for age differences in the evaluation of couple characteristics. Second, we defined a friend in the with-friend condition as one of their best friends regardless of gender because the separation of the condition into heterosexual and same-sex friends could cause inattention to the task due to a long scan time and doing some leisure activities with a heterosexual friend could not be realistic for some soon-to-be-married couples. Additionally, depending on how long a participant has met with the partner or friend, judgment on which leisure activities would be held together could be different, but this factor was not considered in the analysis because no information was obtained about the friend. Therefore, differences in target gender or duration of relationship between the with-friend and with-partner conditions might inevitably affect the results. Third, since the couples were not scanned at the same time, couple characteristics could not be extended to a temporal synchronization and there was no time-dependent analysis of interactions between the couples. Forth, the average participation rate in our behavioral results was about 75% across all conditions. Because of the imbalance between these participating and non-participating responses, we could not analyze the difference in brain response according to the behavioral responses. Fifth, the degree of how exciting participants felt about individual leisure activities was not assessed, and thus no analysis was conducted that reflected such a degree.

In summary, our results revealed both similarities and differences within heterosexual couples in behavioral and neural responses during decisions about participation in leisure activity. Our romantic couples showed decreased activity in the bilateral hippocampus during decisions about participation in leisure activity in the with-partner, maybe because love made episodic memory less useable. Individual leisure activity was correlated with quality of life in the male group, whereas participation in leisure activity might require more cognitive loading on the dorsolateral prefrontal cortex in the female group. The leisure activity matching rate was correlated with courtship period, personality similarity, and spatial similarity of the right frontoparietal network during the task. Our results from the analysis of couples suggest that the spatial similarity of partners’ social brain networks (i.e., the right frontoparietal network) is an important marker that predicts how well a couple enjoys leisure activity together. Moreover, these findings might provide a scientific basis for the saying that couples become more similar the longer they are together. Finally, although partners had different leisure activity patterns, understanding the leisure activity patterns of one’s romantic partner is important for marital satisfaction because soon-to-be married couples will share much of their future lives.

## Material and Methods

### Participants

A total of 42 soon-to-be married heterosexual couples who had no neurological or psychiatric diseases were enrolled in this study. All participants were recruited through advertising on an online wedding community. They were all pre-married couples who have not lived together yet. We paid $70 for each participant in compensation for participation in the experiment, and obtained written informed consent from each participant. The imaging analysis only included data from 37 couples (74 participants) because the data from 5 couples were discarded due to errors in fMRI data acquisition (3 participants) and poor performance (more than 5 missing responses) in the behavioral task (2 participants). The data from the partners of the 5 participants who were excluded from the analysis were also discarded because our study was intended to analyze couples’ responses. This study was approved by the institutional review board of Yonsei University Gangnam Severance Hospital and carried out in accordance with the Declaration of Helsinki.

### Psychological assessments and personality

All participants were asked to answer three different self-report questionnaires before conducting fMRI experiments. An individual’s level of passionate love toward their partner was measured using the passionate love scale (PLS)^60^, which consists of 30 items and 9-point Likert scales. An individual’s personality was measured using the International Personality Item Pool (IPIP), which assesses five factors of personality (neuroticism, extraversion, openness, agreeableness, and conscientiousness) using 10 items per factor^61^. An individual’s quality of life was assessed using the Korean translation of a 26-item, abbreviated version of the World Health Organization Quality of Life (WHOQOL-BREF) assessment^62^.

### Experimental procedure

The soon-to-be-married couples came to the scanning room together with their mates, but each of them was scanned separately. The scanning order between men and women was randomly assigned. Between the scans of the two, there was an interval of about 20 minutes to prepare for scanning, during which time they were instructed not to exchange any information about the experimental task during scanning.

**Figure 5:**
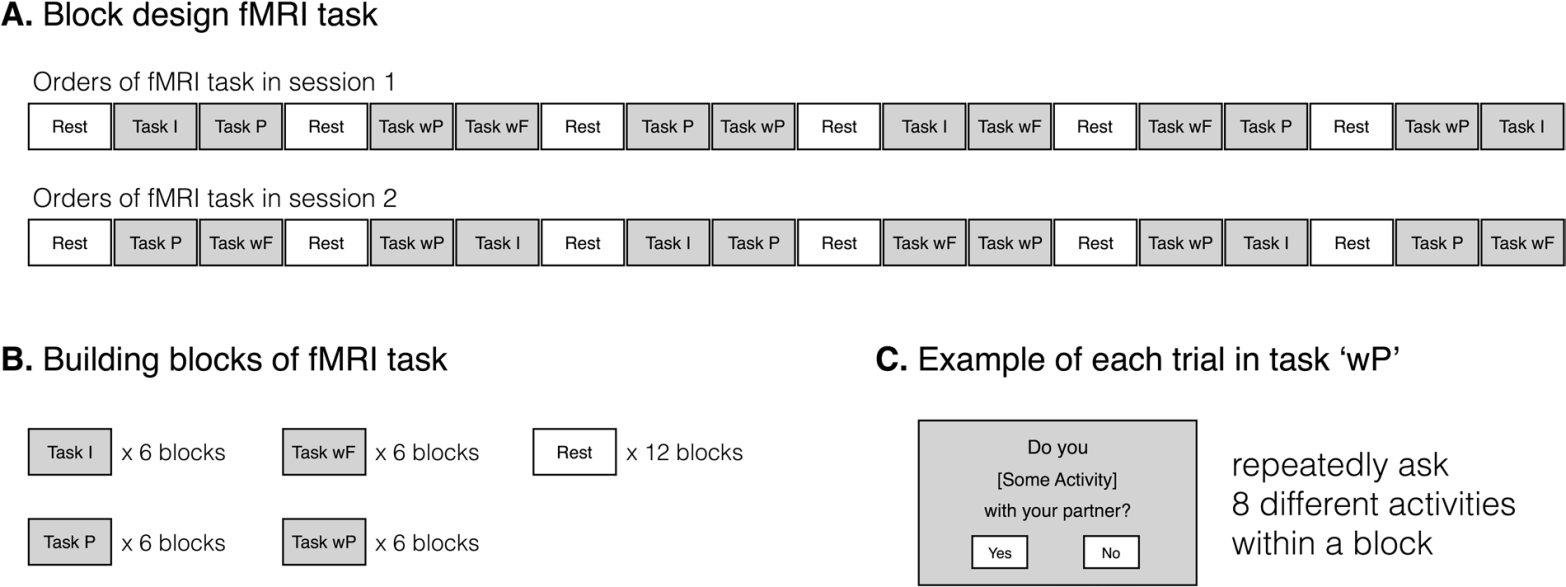
Block-design fMRI task. Diagrams show entire sequences for each session of fMRI task (**A**), building blocks of fMRI task (**B**), and example of each trial in task ‘wP’ (**C**). Abbreviation: I, individual; P, partner; wF, with-friend; wP, with-partner.

Two sessions of a block-design task lasting 6 min each were performed inside the MRI scanner (see Fig. 5 for task design). Each session included 12 experimental blocks and 6 resting blocks, and each block lasted for a duration of 20 sec. All experimental blocks included 8 trials of 2.5 sec, during which a question displayed on the screen. For each experimental block, a series of questions was to ask whether participants perform a given leisure activity, and they were instructed to respond using a button box to the questions. Different types of questions were given depending on the experimental conditions: the individual condition (“Do you do [some activity] alone?”), partner condition (“Does your partner do [some activity] alone?”), with-friend condition (“Do you do [some activity] with your friend?”), and with-partner condition (“Do you do [some activity] with your partner?”). The partner condition was included because decisions in that condition were the basis for decisions in the with-partner condition and because neural activity in that condition needed to be compared with activities in the individual condition and the partner’s individual condition. Since activities together with anyone else can give different fun compared with activities alone, the with-friend condition was included to control the nonspecific elements of such joint activities in the with-partner condition. This method of control has been used in previous romantic love-related fMRI studies^14,15^. Prior to the experiment, participants were informed that a friend in the with-friend condition should be one of their best friends regardless of gender. The 4 experimental conditions were repeated 3 times in each session. As listed in Supplementary Table S1, a total of 48 leisure activities were randomly presented in each experimental condition. The experimental blocks were sequenced in a pseudo-random order in a session, so as to avoid any repetition of the same condition in successive blocks while maximizing the variety of transitions between conditions. During the resting blocks, participants were asked to look at a crosshair on the screen.

### Imaging data acquisition and preprocessing

Images were acquired using a 3 Tesla MRI scanner (Magnetom Verio, Siemens Medical Systems, Erlangen, Germany). For each participant, we acquired two sessions of task fMRI scans using gradient-recall echo-planner imaging (matrix size = 64 × 64, field of view = 240 mm, number of slices = 30, slice order = bottom-up and interleaved, slice thickness = 5 mm, echo time = 30 ms, repetition time = 2,000 ms, flip angle = 90°, bandwidth = 2,232 Hz/Px). Anatomical T1-weighted images were obtained in the sagittal direction using a 3D spoiled-gradient-recall sequence with the following parameters: matrix size = 256 × 256; field of view = 250 mm; number of slices = 176; slice thickness = 1 mm; echo time = 2.46 ms; repetition time = 1,900 ms; flip angle = 9°; bandwidth = 170 Hz/Px.

All fMRI data were preprocessed using Statistical Parametric Mapping 12 (SPM12, http://www.fil.ion.ucl.ac.uk/spm). The first three fMRI scans during the resting state were discarded to allow for magnetization stabilization. The remaining 177 fMRI scans for each session were realigned to the first image of each session and corrected for acquisition time delays between different slices. Then, individual T1-weighted images were co-registered to the mean fMRI image. Subsequently, the fMRI data were spatially normalized using nonlinear transformation functions obtained by deforming individual T1-weighted images to the Montreal Neurological Institute template space, and smoothed with a 6-mm full-width at half-maximum Gaussian kernel.

### Behavioral data analysis

The first variable for behavioral data analysis was leisure activity participation rate, which was defined as the ratio of the number of leisure activities responded with “yes” to the total number of leisure activities presented in each condition. The second variable was reaction time. Two analysis of variance (ANOVA) models were applied to analyze these two variables. In ANOVA model I, we considered sex and two experimental conditions, individual and together, where together was the average of the with-friend condition and the with-partner condition: 2 (sex, male versus female) × 2 (condition, individual versus together). In the post-hoc analysis, we correlated the WHOQOL-BREF scores with the behavioral variables in the individual condition, because the pattern of leisure activity would be associated with an individual’s life satisfaction. In ANOVA model II, we considered sex and the together experimental conditions, with-friend and with-partner: 2 (sex, male versus female) × 2 (condition, with-friend versus with-partner). In the post-hoc analysis, we correlated the PLS scores with the behavioral variables in the with-friend and with-partner conditions. In both ANOVA models, sex was entered as a between-subject factor, and age was entered as a covariate.

### fMRI activation analysis

A general linear model (GLM) was set up by specifying the onset and duration of the task blocks for the four conditions as separate regressors, and the resulting box-car functions were convolved with the canonical hemodynamic response function. High-pass filters (>1/128 Hz) were applied to remove low-frequency scanner drift. After the first-level model estimation, contrast images of the individual, partner, with-friend, with-partner, and together (0.5 × with-friend condition + 0.5 × with-partner) conditions were used to perform a second-level inference. First, paired-sample t-test was conducted to compare neural responses between the individual and partner conditions and two-sample t-test was performed to compare the partner condition and the partner’s individual condition. Then, the contrast images were analyzed with two ANOVA models in the same way as the behavioral data analyses to identify brain regions showing significant main and interaction effects. Statistical inferences were performed at a threshold of the cluster-level family-wise error-corrected P (P_FWE_) < 0.05 with the cluster forming threshold at the voxel level of uncorrected *P* < 0.001. As a post-hoc test, the mean beta values of spheres with a 6-mm radius centered on the peak coordinate in clusters showing significant results were compared between the conditions. These beta values were used for partial correlation analysis with the WHOQOL-BREF or PLS scores in each task condition and each sex while controlling for age. The WHOQOL-BREF scores were correlated with the values from ANOVA model I (condition: individual vs. together) because an individual’s leisure activity has been associated with life satisfaction^7^, whereas the PLS scores were correlated with the values from ANOVA model II (condition: with-friend vs. with-partner) because couples’ shared participation in leisure activity has been associated with relationship quality^63^. In these analyses, the significant correlations were identified after the Bonferroni correction.

### Group independent component analysis and bilateral frontoparietal networks

The preprocessed fMRI data were high-pass filtered to include only frequencies above 1/128 Hz. We then conducted temporal concatenation group ICA (TC-GICA) by following the procedure described in a previous study^64^. Two-step principal component analysis was applied to reduce the preprocessed fMRI data. First, the 354 scans for 2 sessions per participant were reduced to 30 principal components (PCs). The 30 PCs at the first level explained 99.98 ± 0.01% variance of the fMRI scans in each participant. Second, 2,220 temporal components (30 components × 74 participants) were concatenated to 30 PCs and then unmixed with TC-GICA using the Infomax approach^65^. We determined the number of components using the minimum description length criteria to account for correlated samples^66^. Spatial independent component (IC) maps, which represent intra-network functional connectivity, were extracted using the dual regression approach^67^. Finally, given our hypothesis that decisions about participation in some leisure activity would require the involvement of various frontoparietal regions, two spatial IC maps matched for the bilateral frontoparietal networks were identified using the template-matching approach^68^. These two components were compared between the male and female groups using a two-sample t-test while controlling for age. To identify brain regions showing significant group differences, statistical inferences were performed at a threshold of the cluster-level corrected *P*_FWE_ < 0.05.

### Analysis of couples’ characteristics

**Figure 6:**
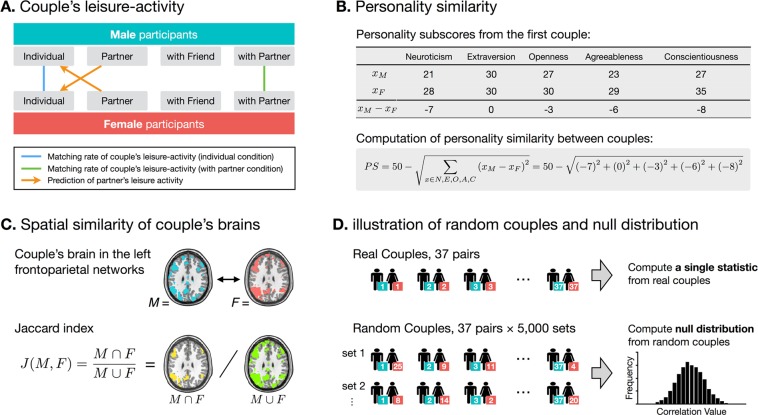
Analysis flow to measure couple characteristics of leisure activity matching rates (**A**), personality similarity (**B**) and spatial similarity of the brains. (**C**) An illustration of random couples and null distribution of random couple characteristics (**D**).

Since our study was conducted on 37 heterosexual couples, we analyzed behavioral and fMRI data by taking advantage of couples’ data. Figure 6 shows the strategy used to extract couple characteristics. To understand them, we computed the similarity of partners’ leisure activity patterns, similarity of their personalities, and spatial similarity of their brains during the leisure activity fMRI task. First, the leisure activity matching rate between the partners in each couple was computed as the ratio of the number of matching leisure activities to the total number of leisure activities in each of the individual and with-partner conditions (Fig. 6A), and the results in the two conditions were compared using a paired-sample t-test. Then, each participant’s prediction accuracy of his/her partner’s leisure activity was computed by matching the participant’s partner condition with his/her partner’s individual condition (Fig. 6A); the results were compared between the male and female groups using paired sample t-test. Additionally, personality similarity between partners within a couple was computed using a concept of Euclidean distance (d) as follows:

$$PS=50-d,{\rm{where}}\,d=\sqrt{\sum _{x\in \{N,E,O,A,C\}}{({x}_{M}-{x}_{F})}^{2}}$$

where PS indicates personality similarity; *x* is a score in five factors of personality including neuroticism (N), extraversion (E), openness (O), agreeableness (A), and conscientiousness (C); and M and F indicate male and female, respectively, for the soon-to-be married couple. An example calculation of personality similarity is shown in Fig. 6B. The Euclidean distance (d) was subtracted from 50 to invert dissimilarity to similarity value, where 50 is the maximum subscore of personality traits. Finally, spatial similarity of the partner’s brains in the bilateral frontoparietal networks was computed using the Jaccard similarity coefficient (*J*). This coefficient was defined as the amount of intersection between the partners’ frontoparietal networks divided by the amount of union between the partners’ frontoparietal networks (Fig. 6C).

We assessed the relationships (1) between length of courtship period and couple characteristics such as personality similarity, leisure activity matching rate, and similarity of the frontoparietal network, (2) between personal similarity and leisure activity matching rate (personality-behavior relationship), and (3) between frontoparietal network similarity and leisure activity matching rate (brain-behavior relationship). These relationships were analyzed using Spearman’s non-parametric partial correlation (*ρ*_couple_) while controlling for age difference within a couple. To test randomness of couple characteristics, we obtained the null distribution from 5,000 sets of random male-female pairs (Fig. 6D). We computed the tail probability (Prob) from the null distribution to determine the significance of couple characteristics at a threshold of *P* < 0.05 for two-side testing (Prob(*ρ* > *ρ*_couple_) or Prob(*ρ* < *ρ*_couple_) < 0.05/2).

## Supplementary information

List of leisure activities
