# Supplementary material for: Neural basis of romantic partners’ decisions about participation in leisure activity
Source: Sci Rep. 2019 Oct 8;9:14448. doi: 10.1038/s41598-019-51038-7 (PMC6783572; doi:10.1038/s41598-019-51038-7)
Supplement: Supplementary file 1 — List of leisure activities [file 41598_2019_51038_MOESM1_ESM.docx]

**Neural basis of romantic partners’ decisions about participation in leisure activity**

Sunghyon Kyeong^1^, Hyojung Eom^2^, Min-Kyeong Kim^1,3^, Young Hoon Jung^2^, Sunyoung Park^1^, and Jae-Jin Kim^1,2,3^

*^1^Institute of Behavioral Science in Medicine, Yonsei University College of Medicine, Seoul, Republic of Korea*

*^2^Brain Korea 21 PLUS Project for Medical Science, Yonsei University, Seoul, Republic of Korea
^3^Department of Psychiatry, Yonsei University College of Medicine, Seoul, Republic of Korea*

**Supplementary Table S1**

A total of 48 leisure activities listed below were presented in a random order during fMRI task

| List of leisure activities |  |
| --- | --- |
| watch TV | babysit |
| play golf | watch baseball |
| walk park | watch a play |
| go shopping for military equipment | watch a movie |
| go fishing | go on expeditions |
| watch news | go on hot spring tours |
| go to the library | go shopping for clothes |
| go to the zoo | yoga |
| watch TV series | cook |
| go hiking | listen to music |
| buy lottery | do online shopping |
| read comic book | eat Chinese food |
| drink beer | ride a bicycle |
| watch motor show | get groceries |
| travel to America | go to exhibitions |
| go to hair salon | go shopping for electronics |
| go backpacking | go jogging |
| go bungee jumping | eat Jokbal |
| get Botox | watch soccer |
| go boxing | go camping |
| eat bulgogi | play computer games |
| travel to Sokcho | Eat cake |
| go to the aquarium | go shopping for cosmetics |
| go skiing | grow plants |
